# Obesity and Hyperandrogenemia in Polycystic Ovary Syndrome: Clinical Implications

**DOI:** 10.3390/jpm13091319

**Published:** 2023-08-28

**Authors:** Mardia López-Alarcón, Víctor Saúl Vital-Reyes, Eduardo Almeida-Gutiérrez, Jorge Maldonado-Hernández, Salvador Flores-Chávez, Juan Manuel Domínguez-Salgado, José Vite-Bautista, David Cruz-Martínez, Aly S. Barradas-Vázquez, Ricardo Z’Cruz-López

**Affiliations:** 1 Unidad de Investigación Médica en Nutrición, Centro Médico Nacional Siglo XXI, Instituto Mexicano del Seguro Social (IMSS), Ciudad de México 06270, Mexico; 2 Departamento de Medicina Reproductiva, Hospital de Ginecología y Obstetricia, Centro Médico Nacional La Raza, Instituto Mexicano del Seguro Social (IMSS), Ciudad de México 02990, Mexico; 3 Departmento de Investigación y Educación en Salud, Hospital de Cardiología, Centro Médico Nacional Siglo XXI, Instituto Mexicano del Seguro Social (IMSS), Ciudad de México 06270, Mexico; 4 Facultad de Medicina, Universidad Autónoma de Nuevo León, Monterrey 64460, Mexico

**Keywords:** polycystic ovary syndrome, obesity-related metabolic disorders, hyperandrogenemia

## Abstract

Polycystic ovary syndrome (PCOS) is often accompanied with metabolic disturbances attributed to androgen excess and obesity, but the contribution of each has not been defined, and the occurrence of metabolic disturbances is usually not investigated. Ninety-nine women with PCOS and forty-one without PCOS were evaluated. The clinical biomarkers of alterations related to glucose (glucose, insulin, and clamp-derived glucose disposal − *M*), liver (aspartate aminotransferase, alanine aminotransferase, and gamma-glutamyl transferase), and endothelium (arginine, asymmetric dymethylarginine, carotid intima-media thickness, and flow-mediated dilation) metabolism were measured; participants were categorized into four groups according to their obesity (OB) and hyperandrogenemia (HA) status as follows: Healthy (no-HA, lean), HA (HA, lean), OB (no-HA, OB), and HAOB (HA, OB). Metabolic disturbances were very frequent in women with PCOS (≈70%). BMI correlated with all biomarkers, whereas free testosterone (FT) correlated with only glucose- and liver-related indicators. Although insulin sensitivity and liver enzymes were associated with FT, women with obesity showed lower M (coef = 8.56 − 0.080(FT) − 3.71(Ob); *p* < 0.001) and higher aspartate aminotransferase (coef = 26.27 + 0.532 (FT) + 8.08 (Ob); *p* = 0.015) than lean women with the same level of FT. Women with obesity showed a higher risk of metabolic disorders than lean women, independent of hyperandrogenemia. Clinicians are compelled to look for metabolic alterations in women with PCOS. Obesity should be treated in all cases, but hyperandrogenemia should also be monitored in those with glucose-or liver-related disturbances.

## 1. Introduction

Many challenges remain in understanding the basis and lack of therapeutic success in the treatment of polycystic ovary syndrome (PCOS), a clinically heterogeneous condition characterized by ovarian dysfunction, hyperandrogenism, and multiple ovarian cysts [1,2]. This is partly because there are still gaps in knowledge about the pathophysiology of the syndrome, but also because patients with PCOS often seek medical attention only if they experience severe clinical manifestations or for reproductive reasons and, consequently, treatment is frequently tailored to ameliorate specific manifestations or to meet requirements of individual needs. Thus, the existence of metabolic conditions commonly associated with the syndrome, such as diabetes, dyslipidemia, and cardiovascular and hepatic diseases [2,3] are usually not investigated. Scientific evidence suggests that androgen excess plays a critical role in the pathophysiology of metabolic disturbances [3,4], but it is unclear whether these disturbances are related to obesity, as PCOS often coexists with this phenotype. Identifying the contribution of obesity and hyperandrogenemia to the risk of clinical metabolic conditions and recognizing the importance of identifying early metabolic alterations is a priority, as this would allow us to take prompt actions to delineate the appropriate therapeutic approach to modify the natural history of the syndrome.

The metabolic morbidity associated with PCOS, including insulin resistance, diabetes, dyslipidemia, and endothelial disturbances that lead to cardiovascular disease, has been extensively evaluated in population-based studies and in several meta-analyses [5,6,7,8,9,10]. Although studies agree that patients with PCOS are at risk for metabolic disorders, almost none of them identify the isolated contribution of androgen excess or obesity. Few studies have made some approximations eliminating the effect of obesity by comparing lean with lean-control PCOS patients [5] or adjusting for nutritional status [6,10], but the results remain inconsistent.

From a clinical perspective, this information is relevant for choosing the appropriate medical treatment. However, to properly assign the detrimental effect to obesity or hyperandrogenemia, it is essential to exclude the influence of other factors also involved in the development of metabolic conditions, such as age, sedentary lifestyles, dyslipidemia, smoking, and the type of lipid intake in the diet [3,11,12,13]. Nevertheless, this is not an easy task, because most of these factors are interrelated.

Under the hypothesis that androgen excess is the main contributor of metabolic alterations in patients with PCOS, in the present study we aimed to separate the contribution of androgen excess and obesity on the susceptibility to develop metabolic disorders in women with PCOS. The contribution of hyperandrogenemia and obesity to the risk of developing metabolic disturbances related to glucose, lipids, endothelium, and liver, was analyzed, considering the influence of important confounders. The frequency of metabolic disorders was compared between women with or without hyperandrogenemia and women with or without obesity.

## 2. Materials and Methods

### 2.1. Design

A cross-sectional study was conducted on patients with PCOS and unrelated controls at hospitals of the Instituto Mexicano del Seguro Social (IMSS) in Mexico City. Participants were enrolled between September 2018 and January 2020. Patients diagnosed with PCOS were recruited at the Department of Reproductive Medicine of a gynecological hospital, the research procedures were carried out at the Medical Nutrition Research Unit, and ultrasound measurements were performed at the Department of Research and Health Education of a cardiology hospital. Women without PCOS were recruited in the waiting rooms of the participating hospitals. The protocol was authorized by the IMSS National Committee for Scientific Research (R-2018-785-101). Written informed consent was obtained from all participants. The study adheres to the STROBE reporting guidelines (https://www.equator-network.org/wp-content/uploads/2015/10/STROBE_checklist_v4_cross-sectional.pdf. (accessed on 8 August 2023)).

Women with or without PCOS according to the Rotterdam criteria [1] were enrolled. Body mass index (BMI) above 30 kg/m^2^ or below 25 kg/m^2^ was used to define obesity and normal weight, respectively. For sampling, women diagnosed with PCOS, with or without obesity, were recruited and, in parallel, an intentional search was performed for women with or without obesity but without the syndrome. Selected women were between 18 and 38 years old, who did not take medication, hormones, or supplements, who were non-smokers, and who did not have diabetes, hypertension, or any known cardiovascular disease. Women without PCOS who were relatives of a patient with PCOS were excluded. Field workers were trained and standardized for all study procedures. The first appointment was arranged to explain the protocol procedures, measure blood pressure and anthropometry, and sign the informed consent form. The second appointment was scheduled within a week at 7:00 am to measure ultrasound variables, apply a 24 h recall questionnaire, and perform a hyperinsulinemic-euglycemic clamp. All the women underwent a pregnancy test before starting the clamp. Fasting blood samples were obtained, centrifuged at 3000 rpm, and serum aliquots were reserved at −20 °C until biochemical determinations.

### 2.2. Measurements

To determine the common carotid intima-media thickness (CIMT), women were placed in a supine position with the neck extended and rotated 45 degrees. The transducer was positioned at an inclination of 45–50 degrees (Samsung Medison, Sonoace R3 model 7.0–13 MHz linear transducer, Seoul, Korea). The measurement point was set in the proximal carotid at 3–4 cm from the bulb with the ultrasound beam directed perpendicularly to the carotid to identify the intima-media. CIMT was measured from the intima-blood interface to the adventitia-media interface [14]. The right and left sides were measured, and the highest value was used for analysis. To determine the flow-mediated dilation (FMD) of the brachial artery, women were placed on their left side with the right arm extended. Blood pressure was measured with a manual sphygmomanometer (Check A Teck by Hergom, B2_D model), the ultrasound transducer was positioned on the right arm, the brachial artery was identified, and the diameter was recorded. Immediately after, the sphygmomanometer cuff was placed 2 cm above the antecubital fold and insufflated for 5 min at 50 mmHg above the systolic blood pressure taken at the beginning. After 60 s of cuff deflation, the artery diameter was measured again. FMD was calculated using the formula: D1−D0/D0 × 100 (where D0 and D1 are the first and second diameters measured) [15].

Multiple-pass 24 h recall questionnaires were collected once to obtain dietary intake information [16]. Nutrient quantification was performed with Food Processor software (v11.7, 2000, ESHA Research Inc., Salem, OR, USA). Energy and nutrient intakes were expressed as absolute values and as the percentage of recommendations [17,18,19]. The women were asked if they were involved in any structured exercise routine, and the expected response was yes or no. If the answer was yes, the time in minutes was registered.

Clamps were conducted as proposed by DeFronzo [20] at 8:00 am after 10 h of fasting. An antecubital venous catheter was placed to administer glucose and insulin infusions. A second retrograde catheter was placed in the opposite hand for sample collection while the hand was kept in a warming device at a temperature of 50–65 °C (Moist Heating Pad, ^®^BesMed: HT-00257, Mexico City, Mexico). Blood samples were drawn at 5 min intervals for glucose determinations (0.5 mL). Insulin infusion started at 80 mU/m^2^ body surface area (BSA) for 10 min, followed by a constant infusion of 40 mU/m^2^ BSA. A 20% glucose solution was administered at a variable rate to maintain plasma glucose at 90 mg/dL. The mean glucose infusion rate (M, mg/kg/min) was assessed during the last 30 min when the steady state (glucose concentration at 90 ± 3 mg/dL) was reached (Figure S1).

Serum glucose, alanine aminotransferase (ALT), aspartate aminotransferase (AST), gamma-glutamyl transferase (GGT), triglycerides, total cholesterol, HDL, and VLDL (Spinreact. Sant Esteve D’en Bas, Spain) were determined via enzymatic analysis (YSI 2300 Stat Plus Glucose Analyzer, YSI Inc., Yellow Springs, OH, USA). LDL was estimated using the Friedewald method [21]. Insulin and steroid hormone-binding globulin (SHBG) were measured via chemiluminescence (Immulite, Siemens; Cambridge, UK), arginine, and asymmetric dimethyl arginine (ADMA) using high-performance liquid chromatography (Waters, ACQUITY UPLC system; Milford, MA, USA), and total testosterone (TT) via UPLC-MS/MS (Waters Xevo TQD Acquity UPLC H; Milford, MA, USA). Free testosterone (FT) was calculated using the Vermeulen method [22]. The coefficients of variation in biochemical assays ranged from 5–10%.

### 2.3. Diagnosis of Metabolic Disorders

The cut-off points used for diagnosis were as follows: M < 5.7 mg/kg/min for insulin resistance [23]; fasting glucose > 100 mg/dL for prediabetes [24]; ADMA > 0.88 μmol/L [25]; CIMT > 0.5 mm [14] or FMD < 10% [15] for endothelial dysfunction; ALT > 30 U/L [26] or AST: ALT ratio < 1.0 for liver damage [27]; non-HDL > 144 mg/dL for dyslipidemia [13]; and blood pressure > 120/80 for hypertension [28]. FT > 5.6 pg/mL was used to identify hyperandrogenemia [29] and women were classified with obesity if BMI ≥ 30 kg/m^2^, or without obesity if BMI ≤ 25 kg/m^2^ [30].

### 2.4. Statistical Analysis

To calculate the sample size, we used the information reported by Pradisi et al. [31], who evaluated lipid profile, insulin, and endothelial function in women with and without PCOS with obesity. A correlation coefficient between leg blood flow and free testosterone −0.52, 80% beta, 0.05 alpha, as well as a mean lipoprotein difference of 8 mg/dL between overweight women with and without PCOS were used. The calculated sample size was 51 women with PCOS and 51 without PCOS. Forty women were added to conduct a stratified analysis according to obesity and hyperandrogenemia status. The estimated sample size was 142 women.

The Minitab statistical package (v19, State College, PA, USA) was used for the analysis. A *p*-value ≤ 0.05 was considered statistically significant. Quantitative variables were expressed as means, standard errors (SEM) or 95% confidence intervals (95%CI), and qualitative variables as proportions. The equality of variances was analyzed using Levene’s test. Differences between two independent groups (PCOS vs. non-PCOS) were analyzed using the Student *t*-test. Pearson correlation analyses were carried out to identify univariate associations between biomarkers. Associations among qualitative variables were evaluated via χ^2^ analyses.

For multiple analyses, regression models were carried out considering metabolic biomarkers as dependent variables, BMI and FT as predictors, and age and dietary lipids (total lipids, saturated fat, trans, ω-3, and 6 fatty acids) intake as confounders; the presence of PCOS and dyslipidemia were introduced as covariates. The General Linear Model approach was used to examine the fixed effects of obesity and hyperandrogenemia on metabolic markers, and Bonferroni post hoc test and interactions were evaluated. The variance inflation factor was used to evaluate collinearity. In another statistical approach, multiple regression analyses were conducted to explore the association between FT and biomarkers, introducing obesity as a covariate.

To separate the influence of obesity (OB) and hyperandrogenemia (HA) on the metabolic disorders studied, women were categorized into four groups: Healthy (no-HA, lean), HA (HA, lean), OB (no-HA, OB) and HAOB (HA and OB). The risk of glucose-, lipid-, endothelial-, and liver-related disorders was assessed via logistic regression analysis, introducing the stratified groups as predictors. Models were adjusted for age, dyslipidemia, and dietary lipids (total lipids, saturated fat, trans, ω-3, and 6 fatty acids).

## 3. Results

### 3.1. General Characteristics

We studied 140 women aged 29 ± 5.4 years, with a BMI of 30.4 ± 6.4 kg/m^2^. Daily energy intake was 2163 ± 80 kcal, sugar 88 ± 5 g/d, total lipids 71 ± 4 g/d, saturated fat 22 ± 1 g/d, and omega-3 fatty acids 0.9 ± 0.1 g/d (Table S1: Dietary intake). None of the women reported a structured exercise routine. As expected, significant interrelations were observed between the variables studied in the simple correlation analyses (Table S2: Univariate correlations). Ninety-nine women met the diagnostic criteria for PCOS.

**Figure 1:**
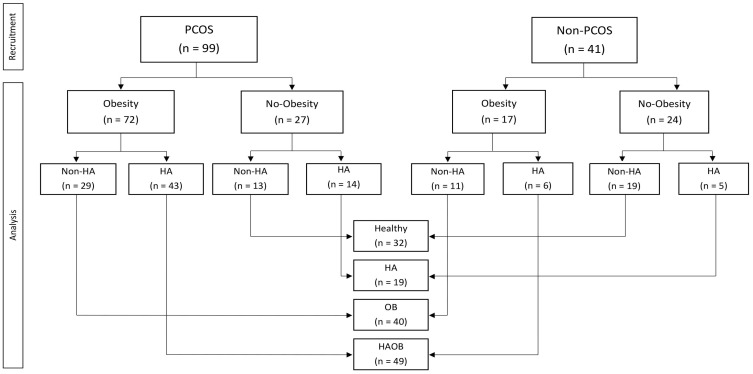
Flow diagram and stratification.

The average of most measurements in patients with PCOS was different from that of controls and was outside the reference limits (Table S3: Clinical and biochemical characteristics of women with or without PCOS). The frequency of insulin resistance (68% vs. 22%), dyslipidemia (32% vs. 12%), endothelial dysfunction (79% vs. 61%), carotid intima-media thickening (58% vs. 39%), and biomarkers of hepatic disturbances (89% vs. 63%) were significantly higher in the groups of women with PCOS than in those without PCOS. Mean (±SE) BMI (31.77 ± 0.59 vs. 27.18 ± 1.0 kg/m2, *p* < 0.001) and free testosterone (8.2 ± 0.60 vs. 4.6 ± 0.63 pg/mL, *p* < 0.001) were also higher in women with PCOS than in those without the syndrome. Obesity was observed in 72 (73%) women with PCOS and 17 (42%) without PCOS, and hyperandrogenemia in 57 (58%) and 11 (27%) women, respectively (Figure 1).

### 3.2. Associations between Free Testosterone and BMI with Biomarkers of Metabolic Disorders

Adjusted linear models showed that BMI was related to almost all metabolic biomarkers, whereas FT was only associated with insulin and liver-related biomarkers. Insulin sensitivity decreased further in patients with PCOS (Table 1), whereas dietary *ω*-3 fatty acids increased it (M = 0.744 ± 0.246, *p* = 0.003). The introduction of confounders did not modify the associations of BMI and FT with biomarkers.

**Figure 2:**
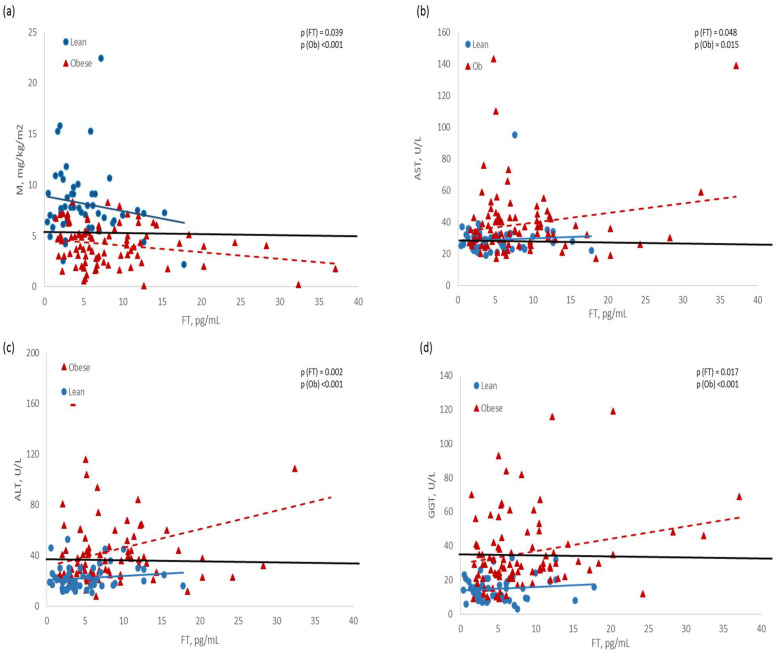
Linear regression models were used to analyze the associations of free testosterone (FT) with M (**a**), aspartate aminotransferase (**b**), alanine aminotransferase (**c**), and glutamyl aminotransferase (**d**). Horizontal solid lines represent the reference values for normality: M > 5.7 mg/kg/min [23]; AST < 31 U/L [32]; ALT < 30 U/L [26]; GGT < 40 U/L [32]. Women were classified as obese if BMI ≥ 30 kg/m^2^, or lean if BMI ≤ 25 kg/m^2^ [30]; those with obesity showed significantly different slopes compared to the lean women.

With another statistical approach, we observed that FT was inversely associated with clamp-derived glucose disposal (M), and directly associated with liver enzymes, but women with obesity had lower M values and higher enzyme concentrations than women without obesity at the same level of FT. The regression line of women without obesity was mostly within normal ranges (Figure 2).

### 3.3. Contribution of Obesity and Hyperandrogenemia to the Susceptibility of Metabolic Disorders

Most clinical and biochemical variables were affected by obesity, whereas hyperandrogenemia affected only blood pressure and insulin concentration. The dietary intake of trans fatty acids tended to be higher (*p* = 0.076), and that of omega-3 (*p* = 0.001) and omega-6 (*p* = 0.042) fatty acids was lower in groups with obesity than in those without obesity (Table 2). All the other dietary variables were comparable among groups.

The studied biomarkers were compared among 32 Healthy, 19 HA, 40 OB, and 49 HAOB women. Hypertension (8%) and prediabetes (11%) were observed exclusively in the two groups with obesity. Overall, the proportion of women with measurements outside the reference limits was higher in the two groups with obesity than in those without it. Consequently, the risk of metabolic disturbances was several orders of magnitude higher in the OB and HAOB groups than in the Healthy (control) group, and the risk of having M, HDL, CIMT, and liver enzymes outside normal ranges (described in Section 2.3: Diagnoses of Metabolic Disorders) in women with obesity was also higher compared to women with hyperandrogenemia but without obesity (HA). Susceptibility to metabolic disturbances was comparable between the OB and HAOB groups (Table 3).

## 4. Discussion

In the present study, we demonstrated that obesity is the main factor that contributes to the development of metabolic disorders in patients with PCOS. However, we also identified a mild but consistent effect of androgens on glucose- and liver-related biomarkers that were independent of obesity. We also confirmed the high frequency of metabolic disorders in women with PCOS, as already reported by others [2,5,8,9,10], and additionally, provided data concerning the high frequency of metabolic alterations that have not yet been revealed as a pathological entity.

Although the detrimental effect of both obesity and androgen excess in patients with PCOS has already been reported [2,3,4], our analysis allowed us to discriminate the contribution of each to the risk of metabolic disturbances, which is paramount to choosing the appropriate therapeutic approach. This analysis revealed that the influence of obesity is stronger than that of androgen excess, as the two groups with obesity showed comparable risks of metabolic disturbances independently of hyperandrogenemia, and higher values than those without obesity. Such results show the predominant involvement of obesity in the development of metabolic diseases in patients with PCOS. Nevertheless, since the group of women with hyperandrogenemia and normal weight consistently showed intermediate risks between healthy and OB, the involvement of androgen excess in the susceptibility to metabolic disturbances could not be ruled out. Indeed, the potential influence of androgens on metabolism was observed in the linear regression models, which demonstrated a dose–response association between androgens and glucose- and liver-related biomarkers. That is, the higher the FT concentration, the lower the M and the higher the concentration of the hepatic enzyme, even within normal ranges. We interpret these results as evidence of a mild but consistent influence of androgens on metabolism, which may progress toward a pathological condition.

Our study confirmed the high frequency of clinical conditions in patients with PCOS, including dyslipidemia, thickened carotid, and liver alterations, reported in other studies [2,5,8,9,10]. We also provided additional evidence regarding the high frequency of metabolic alterations, such as decreased insulin sensitivity and increased biomarkers of endothelial and hepatic alterations, which have not reached the cut-off point to diagnose a clinical condition but are precursors of chronic pathologies. This is clinically relevant because it underlies the need to intentionally search for these disturbances to prevent progression to an irreversible condition such as diabetes, endothelial dysfunction, and non-alcoholic fatty liver disease (NAFLD).

We acknowledge some limitations, mainly the small sample of women in the HA group. Although this circumstance may lead to a lack of power to detect differences between the groups as observed in the stratified analysis, other statistical approaches used in our study, which used continuous data instead of stratification, allowed us to identify the androgenic effect with reasonable certainty. It is also an important limitation that we considered liver enzymes as potential markers of hepatic disturbances. We are aware that these enzymes raise non-specifically in response to liver aggressions from different etiology (e.g., metabolic, viral, toxic, and alcoholic), and even in response to extrahepatic injuries; therefore, their elevation is not a specific indication of metabolic liver damage. Nevertheless, considering the high frequency of obesity-associated hepatic alterations reported in the literature, and that most of our participants exhibited obesity and did not acknowledge alcohol consumption, we think that the liver involvement in these patients is probably high. In addition, we believe that the use of a proposed ALT cut-off point to identify healthy individuals, derived from a huge sample of women without risk factors for liver disease, and validated for its ability to predict liver damage [26], increases the likelihood that our result will be correctly interpreted. Furthermore, the association of liver enzymes with obesity and hyperandrogenemia was consistent throughout the different analyses, suggesting the plausibility of our interpretation. Yet, we recognize that this finding needs to be replicated with appropriate diagnostic tools for liver damage. Finally, we acknowledge that we studied a particular selected population, as participants were recruited from a hospital that treats fertility cases and were purposefully selected if they met the selection criteria until the proposed sample size was completed. Therefore, our results may apply only to women with similar characteristics.

Our study has important strengths that are worth highlighting. The stratified analysis allowed us to disentangle the role of obesity and hyperandrogenemia, providing strong evidence for the predominant role of obesity over that of androgen excess. This is clinically relevant because it provides the basis for selecting the most appropriate therapeutic approach. In addition, we used cutting-edge technology to measure important variables, thus obtaining accurate data on insulin sensitivity (clamp), testosterone (mass-spectrometry), and endothelial dysfunction (two biochemical, ADMA and arginine, and two ultrasound, CIMT and FMD), which improved the chance of detecting any possible effects. Moreover, we excluded overweight women to increase the likelihood of identifying differences in the metabolic profiles of women with or without obesity. Furthermore, we considered the influence of important confounders like dyslipidemia and dietary lipids, which are often associated with the risk of metabolic disorders but are not usually considered in similar studies. We believe that all these characteristics made our results robust and reliable.

The recommendations of the American College of Obstetricians and Gynecologists for the management of patients with PCOS include the evaluation of BMI, blood pressure, and laboratory documentation of hyperandrogenemia and metabolic abnormalities, such as a two-hour glucose tolerance test, as well as fasting lipid and lipoprotein concentrations [33]. Our results support these recommendations and, additionally, provide enough information to encourage intentional screening for liver abnormalities. We are certain that providing specific recommendations for the management of PCOS is beyond the scope of our study, yet it is important to point out the importance of treating obesity and its complications, such as insulin resistance. In this regard, one issue that deserves attention is the effect of a low intake of omega-3 polyunsaturated fatty acids on insulin sensitivity observed in our study. Although the dietary information obtained with a single 24 h recall questionnaire constitutes a limitation for interpreting the influence of nutrient intake, our results are consistent with others found in studies conducted in Mexico by our team [12] and by others [34], suggesting that the extremely low intake of these fatty acids in Mexican population is a reliable circumstance. The issue is important because evidence from experimental studies demonstrates that the long-chain polyunsaturated fatty acids omega-3 are involved in insulin sensitivity and endothelial and hepatic protection [35,36]. In this regard, we had previously reported that eicosapentaenoic acid in erythrocytes, and supplementation with docosahexaenoic and eicosapentaenoic acid, were associated with an improved androgenic profile of pubertal girls with obesity [37]. We should take this into account when planning the treatment of patients with PCOS. On the other hand, the lack of association between metabolic alterations and saturated fat intake is probably explained by the close-to-recommended intake of these nutrients.

## 5. Conclusions

In conclusion, our results have potential clinical implications in the choice of treatment for patients with PCOS, as well as in the prevention of metabolic disturbances and progression to irreversible conditions. Our study also highlights the need for a major effort to improve the detection of metabolic disturbances in clinical settings. Focusing on these situations will hopefully help clinicians and health authorities to make better decisions in the management of PCOS patients to limit the progression to irreversible metabolic conditions.

## Figures and Tables

**Table 1:** BMI and free testosterone as predictors of metabolic biomarkers in 140 women with and without PCOS *.

	BMI, kg/m^2^	FT, pg/mL	Dyslipidemia	PCOS
	Coefficients ± SE			
Glucose, mg/dL	0.562 ± 0.139 ^a^	−0.191 ± 0.146	1.00 ± 1.9	1.04 ± 1.97
Insulin, µU/mL	0.554 ± 0.141 ^a^	0.557 ± 0.150 ^a^	4.74 ± 1.94 ^b^	3.54 ± 1.99 ^d^
M, mg/kg/min	−0.231 ± 0.036 ^a^	−0.050 ± 0.039	−0.62 ± 0.50	−1.66 ± 0.52 ^b^
TG, mg/dL	2.84 ± 1.05 ^b^	1.33 ± 1.12	75.0 ± 14.4 ^a^	−3.3 ± 15
TC, mg/dL	0.146 ± 0.337	0.273 ± 0.360	56.60 ± 4.62 ^a^	−1.76 ± 4.77
LDL, mg/dL	0.106 ± 0.314	−0.053 ± 0.336	42.91 ± 4.31 ^a^	1.19 ± 4.45
VLDL, mg/dL	0.588 ± 0.210 ^b^	0.267 ± 0.224	15.08 ± 2.87 ^a^	−0.30 ± 2.97
HDL, mg/dL	−0.527 ± 0.128 ^a^	0.061 ± 0.137	−1.30 ± 1.76	−2.29 ± 1.82
ADMA, µmol/mL	0.014 ± 0.005 ^b^	−0.008 ± 0.005	0.073 ± 0.061	0.062 ± 0.063
CIMT, mm	0.005 ± 0.003 ^d^	−0.003 ± 0.003	0.016 ± 0.038	0.040 ± 0.039
FMD, %	−0.315 ± 0.242	0.284 ± 0.258	−3.18 ± 3.31	0.24 ± 3.43
AST, U/L	0.276 ± 0.260	0.497 ± 0.277 ^d^	4.39 ± 3.56	3.73 ± 3.68
ALT, U/L	0.778 ± 0.390 ^c^	1.184 ± 0.416 ^b^	7.85 ± 5.35	6.57 ± 5.53
GGT, U/L	0.958 ± 0.257 ^a^	0.626 ± 0.275 ^b^	14.16 ± 2.59 ^a^	−1.53 ± 3.54

* Multivariate linear regression analysis. Models were adjusted by age, dietary lipids intake, dyslipidemia, and PCOS. ^a^ *p* < 0.001, ^b^ *p* <0.01, ^c^ *p* < 0.05, and ^d^ *p* < 0.10. BMI, body mass index; FT, free testosterone; M, clam-derived glucose disposal; TG, triglycerides; TC, total cholesterol; LDL, low-density lipoprotein; VLDL, very low-density lipoprotein; HDL high-density lipoprotein; ADMA asymmetric dimethyl arginine; CIMT, carotid intima-media thickness; FMD, flow-mediated dilation; AST, aspartate aminotransferase; ALT, alanine aminotransferase; GGT, gamma-glutamyl transferase.

**Table 2:** Clinical, anthropometric, biochemical, and ultrasound characteristics of participants, stratified by hyperandrogenic and obesity status (*n* = 140) *.

	Non-Obesity, *n* = 51		Obesity, *n* = 89				
	Healthy, *n* = 32	HA, *n* = 19	OB, *n* = 40	HAOB, *n* = 49	P_Ob_	P_HA_	*P_interaction_*
Clinical							
Age, y	28 ± 1	26 ± 1	31 ± 1	28 ± 1	<0.001	0.076	0.825
SBP, mmHg	102 ± 2	102 ± 2	110 ± 2	115 ± 1	<0.001	0.073	0.141
DBP, mmHg	68 ± 2	69 ± 2	73 ± 1	77 ± 1	<0.001	0.047	0.559
Anthropometric							
BMI, kg/m^2^	23.6 ± 0.6	23.1 ± 0.8	33.9 ± 0.6	34.9 ± 0.5	<0.001	0.457	0.246
BF, %	34.4 ± 0.9	32.7 ± 1.2	44.7 ± 0.8	45.6 ± 0.7	<0.001	0.939	0.150
WHR	0.90 ± 0.03	0.98 ± 0.03	0.93 ± 0.02	0.91 ± 0.02	0.706	0.682	NC
Biochemical							
Glucose, mg/dL	81.9 ± 1.7	83.5 ± 2.2	90.9 ± 1.5	88.7 ± 1.4	<0.001	0.606	0.273
Insulin, μU/mL	6.4 ± 1.8	6.5 ± 2.3	14.5 ± 1.6	21.0 ± 1.2	<0.001	0.017	0.085
M, mg/g/min	8.2 ± 0.5	8.1 ± 0.6	4.4 ± 0.3	4.0 ± 0.4	<0.001	0.474	0.682
TG, mg/dL	103 ± 12	102 ± 14	160 ± 10	177 ± 10	<0.001	0.136	0.372
TC, mg/dL	146 ± 6	164 ± 8	168 ± 5	173 ± 5	0.006	0.109	0.262
LDL, mg/dL	83 ± 5	98 ± 6	102 ± 5	99 ± 4	0.054	0.503	0.078
VLDL, mg/dL	20 ± 3	20 ± 4	30 ± 2	37 ± 2	<0.001	0.115	0.280
HDL, mg/dL	42 ± 2	46 ± 2	36 ± 1	37 ± 1	<0.001	0.214	0.569
Non-HDL, mg/dL	103 ± 6	119 ± 8	133 ± 5	136 ± 5	<0.001	0.185	0.303
AST, U/L	28 ± 3	31 ± 4	40 ± 3	38 ± 3	0.003	0.830	0.371
ALT, U/L	21 ± 5	25 ± 6	45 ± 4	43 ± 4	<0.001	0.912	0.616
GGT, U/L	14 ± 3	15 ± 4	33 ± 3	38 ± 3	<0.001	0.220	0.499
AST/ALT	1.44 ± 0.06	1.31 ± 0.08	1.06 ± 0.06	0.96 ± 0.05	<0.001	0.067	0.861
SHBG, mmol/L	46 ± 3	32 ± 4	34 ± 3	22 ± 2	0.001	<0.001	0.808
TT, ng/dL	19 ± 3	48 ± 4	21 ± 3	51 ± 3	0.347	<0.001	0.881
FT, pg/mL	2.7 ± 0.8	9.0 ± 1.0	3.8 ± 0.7	12 ± 0.6	0.013	<0.001	0.255
ADMA, μmol/L	0.94 ± 0.05	0.99 ± 0.07	1.23 ± 0.05	1.12 ± 0.04	<0.001	0.315	0.137
Arginine/ADMA	71 ± 4	78 ± 5	63 ± 4	69 ± 3	0.032	0.120	0.895
Ultrasound							
CIMT, mm	0.55 ± 0.03	0.53 ± 0.04	0.64 ± 0.03	0.63 ± 0.03	0.007	0.654	0.895
FMD, %	26 ± 4	23 ± 3	16 ± 2	23 ± 2	0.114	0.219	0.185
Dietary							
Lipids, g/d	68 ± 8	84 ± 10	74 ± 7	66 ± 6	0.596	0.993	0.111
Saturated fat, g/d	20 ± 3	28 ± 4	22 ± 2	21 ± 2	0.575	0.533	0.075
Trans, g/d	0.36 ± 0.14	0.37 ± 0.18	0.65 ± 0.12	0.58 ± 0.11	0.076	0.774	0.803
Omega-6, g/d	10.4 ± 1.2	11.7 ± 1.6	9.1 ± 1.1	7.5 ± 1.0	0.042	0.606	0.236
Omega-3, g/d	1.1 ± 0.2	1.5 ± 0.2	0.7 ± 0.1	0.7 ± 0.1	0.001	0.400	0.200

* Mean ± SEM. General linear model analysis. HA, hyperandrogenemia; OB, obesity; OBHA, obesity + hyperandrogenemia; SBP, systolic blood pressure; DBP, diastolic blood pressure; BMI, body mass index; BF, body fat; WHR, waist-to-hip ratio; M-value, clamp-derived glucose disposal; TG, triglycerides; TC, total cholesterol; LDL, low-density lipoprotein cholesterol; VLDL, very low-density lipoprotein cholesterol; HDL, high-density lipoprotein cholesterol; AST, aspartate aminotransferase; ALT, alanine aminotransferase; SHBG, steroid hormone-binding globulin; TT, total testosterone; FT, free testosterone; ADMA, asymmetric dimethyl arginine; CIMT, carotid intima-media thickness; FMD, flow-mediated dilation.

**Table 3:** Separate influence of obesity and hyperandrogenemia on the risk of metabolic disturbances *^,†^.

	Glucose and Lipid-Related Disturbances							
	Cases (%); OR (95% CI)							
	**M-Value < 5.7 mg/kg/min**		**Triglycerides > 150 mg/dL**		**HDL < 50 mg/dL**		**Non-HDL > 144 mg/dL**	
Healthy	4 (13)	1.00 ^‡^	5 (15)	1.00 ^‡^	25 (78)	1.00	2 (6)	1.00
HA	3 (16)	1.31 (0.26, 6.6)	5 (26)	1.93 (0.48, 7.8)	12 (63)	0.48 (0.14, 1.68)	5 (26)	5.36 (0.92, 31)
OB	29 (73)	18.5 (5.25, 64.8)	17 (43)	3.99 (1.27, 12.5)	38 (95)	5.32 (1.02, 28)	12 (30)	6.43 (1.32, 31)
HAOB	40 (82)	31.1 (8.71, 111)	25 (51)	5.63 (1.86, 17)	46 (94)	4.29 (1.02, 18)	18 (37)	8.71 (1.86, 41)
HA	3 (16)	1.00	5 (26)	1.00	12 (63)	1.00	5 (26)	1.00
OB	29 (73)	14.1 (3.41, 57.9)	17 (43)	2.07 (0.63, 6.86)	38 (95)	11.1 (2.02, 60.7)	12 (30)	1.20 (0.35, 4.08)
HAOB	40 (82)	23.7 (5.68, 99)	25 (51)	2.92 (0.91, 9.35)	46 (94)	8.94 (2.01, 40.0)	18 (37)	1.63 (0.50, 5.26)
OB	29 (73)	1.00	17 (43)	1.00	38 (95)	1.00	12 (30)	1.00
HAOB	40 (82)	1.69 (0.62, 4.59)	25 (51)	1.41 (0.61, 3.27)	46 (94)	0.81 (0.13, 5.08)	18 (37)	1.36 (0.56, 3.30)
OB	29 (73)	1.00	17 (43)	1.00	38 (95)	1.00	12 (30)	1.00
HAOB	40 (82)	1.69 (0.62, 4.59)	25 (51)	1.41 (0.61, 3.27)	46 (94)	0.81 (0.13, 5.08)	18 (37)	1.36 (0.56, 3.30)
	Endothelial-Related Disturbances ^‡^							
	**ADMA > 0.88 µmol/L**		**CIMT > 5 mm**		**FMD < 10%**		**Arginine/ADMA < 78**	
Healthy	17 (53)	1.00	13 (41)	1.00	6 (19)	1.00	20 (63)	1.00
HA	13 (68)	1.83 (0.55, 6.11)	5 (26)	0.53 (0.15, 1.86)	3 (16)	0.82 (0.18, 3.79)	11 (61)	0.91 (0.27, 3.02)
OB	33 (83)	3.96 (1.33, 11.8)	27 (68)	3.10 (1.16, 8.33)	13 (33)	2.13 (0.69, 6.60)	32 (80)	2.30 (0.79, 6.74)
HAOB	40 (82)	3.67 (1.30, 10.4)	28 (57)	2.00 (0.78, 5.12)	8 (16)	0.87 (0.26, 2.90)	36 (74)	1.57 (0.58, 4.24)
HA	13 (68)	1.00	5 (26)	1.00	3 (16)	1.00	11 (61)	1.00
OB	33 (83)	2.16 (0.61, 7.67)	27 (68)	5.84 (1.73, 19.7)	13 (33)	2.60 (0.64, 10.5)	32 (80)	2.54 (0.75, 8.64)
HAOB	40 (82)	2.01 (0.60, 6.75)	28 (57)	3.77 (1.17, 12.2)	8 (16)	1.08 (0.25, 4.56)	36 (74)	1.76 (0.56, 5.51)
OB	33 (83)	1.00	27 (68)	1.00	13 (33)	1.00	32 (80)	1.00
HAOB	40 (82)	0.93 (0.31, 2.77)	28 (57)	0.65 (0.27, 1.55)	8 (16)	0.41 (0.15, 1.13)	36 (74)	0.68 (0.25, 1.87)
	Liver-Related Disturbances ^‡^							
	**AST > 31 U/L**		**ALT > 30 U/L**		**GGT > 32 U/L**		**AST/ALT ratio < 1.00**	
Healthy	8 (25)	1.00	2 (6)	1.00	0 (0)	NA	3 (9)	1.00
HA	6 (32)	1.16 (0.32, 4.18)	4 (21)	3.29 (0.53, 21)	1 (5)		4 (21)	2.15 (0.41, 11)
OB	23 (56)	3.41 (1.21, 9.62)	20 (50)	12.5 (2.59, 60)	16 (41)		18 (45)	6.62 (1.70, 26)
HAOB	30 (63)	3.98 (1.44, 11)	30 (61)	19.0 (3.99, 90)	19 (40)		29 (60)	11.87(3.10, 45)
HA	6 (32)	1.00	4 (21)	1.00	1 (5)	1.00	4 (21)	1.00
OB	23 (56)	2.95 (0.91, 9.52)	20 (50)	3.81 (1.05, 13.8)	16 (41)	13.3 (1.6, 111.7)	18 (45)	3.08 (0.85, 11.1)
HAOB	30 (63)	3.44 (1.09, 10.9)	30 (61)	5.77 (1.63, 20.5)	19 (40)	11.58 (1.4, 95.6)	29 (60)	5.52 (1.56, 19.5)
OB	23 (56)	1.00	20 (50)	1.00	16 (41)	1.00	18 (45)	1.00
HAOB	30 (63)	1.17 (0.49, 2.79)	30 (61)	1.52 (0.64, 3.59)	19 (40)	0.87 (0.36, 2.12)	29 (60)	1.87 (0.80, 4.37)

* Logistic regression analysis. ^†^ Models were adjusted by dietary intake of lipids, saturated fat, and ω-3 and ω-6 fatty acids. ^‡^ Models were additionally adjusted by dyslipidemia. *M*, clam-derived glucose uptake; HDL, high-density lipoprotein; ADMA, asymmetric dymethilarginine; CIMT, carotid intima-media thickness; FMD flow-mediated dilation; AST, aspartate aminotransferase; ALT alanine aminotransferase; GGT, gamma-glutamyl transferase. Healthy (no-hyperandrogenemia, lean), HA (hyperandrogenemia, lean), OB (no-hyperandrogenemia, obesity), and HAOB (hyperandrogenemia and obesity).

## Data Availability

The data presented in this study are available upon request from the corresponding authors.

## Supplementary Materials

The following supporting information can be downloaded at: https://www.mdpi.com/article/10.3390/jpm13091319/s1, Table S1: Dietary intake; Table S2: Univariate correlations; Table S3: Clinical and biochemical characteristics. Figure S1: Glucose concentration during clamp procedure. The steady state was reached at the last 30 min.

## References

[1] (2004). Rotterdam ESHRE/ASRM-sponsored PCOS consensus workshop group Revised 2003 consensus on diagnostic criteria and long-term health risks related to polycystic ovary syndrome (PCOS). Hum. Reprod..

[2] American College of Obstetricians and Gynecologists’ Committee on Practice Bulletins—Gynecology (2018). ACOG Practice Bulletin No. 194: Polycystic Ovary Syndrome. Obstet. Gynecol..

[3] Moghetti P., Tosi F., Bonin C., Di Sarra D., Fiers T., Kaufman J.-M., Giagulli V.A., Signori C., Zambotti F., Dall’Alda M. (2013). Divergences in Insulin Resistance Between the Different Phenotypes of the Polycystic Ovary Syndrome. J. Clin. Endocrinol. Metab..

[4] Kakoly N.S., Earnest A., Teede H.J., Moran L.J., Joham A.E. (2019). The Impact of Obesity on the Incidence of Type 2 Diabetes Among Women With Polycystic Ovary Syndrome. Diabetes Care.

[5] Zhu S., Zhang B., Jiang X., Li Z., Zhao S., Cui L., Chen Z.-J. (2019). Metabolic disturbances in non-obese women with polycystic ovary syndrome: A systematic review and meta-analysis. Fertil. Steril..

[6] Ollila M.-M., West S., Keinänen-Kiukaanniemi S., Jokelainen J., Auvinen J., Puukka K., Ruokonen A., Järvelin M.-R., Tapanainen J., Franks S. (2017). Overweight and obese but not normal weight women with PCOS are at increased risk of Type 2 diabetes mellitus—A prospective, population-based cohort study. Hum. Reprod..

[7] Zhou Y., Wang X., Jiang Y., Ma H., Chen L., Lai C., Peng C., He C., Sun C. (2017). Association between polycystic ovary syndrome and the risk of stroke and all-cause mortality: Insights from a meta-analysis. Gynecol. Endocrinol..

[8] Wekker V., van Dammen L., Koning A., Heida K.Y., Painter R.C., Limpens J., Laven J.S.E., Lennep J.E.R.v., Roseboom T.J., Hoek A. (2020). Long-term cardiometabolic disease risk in women with PCOS: A systematic review and meta-analysis. Hum. Reprod. Update.

[9] Rocha A.L.L., Faria L.C., Guimarães T.C.M., Moreira G.V., Cândido A.L., Couto C.A., Reis F.M. (2017). Non-alcoholic fatty liver disease in women with polycystic ovary syndrome: Systematic review and meta-analysis. J. Endocrinol. Investig..

[10] Sarkar M., Terrault N., Chan W., Cedars M.I., Huddleston H.G., Duwaerts C.C., Balitzer D., Gill R.M. (2020). Polycystic ovary syndrome (PCOS) is associated with NASH severity and advanced fibrosis. Liver Int..

[11] Nettleton J.A., Villalpando S., Cassani R.S.L., Elmadfa I. (2013). Health Significance of Fat Quality in the Diet. Ann. Nutr. Metab..

[12] López-Alarcón M., Perichart-Perera O., Flores-Huerta S., Inda-Icaza P., Rodríguez-Cruz M., Armenta-Álvarez A., Bram-Falcón M.T., Mayorga-Ochoa M. (2014). Excessive Refined Carbohydrates and Scarce Micronutrients Intakes Increase Inflammatory Mediators and Insulin Resistance in Prepubertal and Pubertal Obese Children Independently of Obesity. Mediat. Inflamm..

[13] Brunner F.J., Waldeyer C., Ojeda F., Salomaa V., Kee F., Sans S., Thorand B., Giampaoli S., Brambilla P., Tunstall-Pedoe H. (2019). Application of non-HDL cholesterol for population-based cardiovascular risk stratification: Results from the Multinational Cardiovascular Risk Consortium. Lancet.

[14] Touboul P.-J., Grobbee D.E., Ruijter H.D. (2012). Assessment of subclinical atherosclerosis by carotid intima media thickness: Technical issues. Eur. J. Prev. Cardiol..

[15] Yeboah J., Folsom A.R., Burke G.L., Johnson C., Polak J.F., Post W., Lima J.A., Crouse J.R., Herrington D.M. (2009). Predictive Value of Brachial Flow-Mediated Dilation for Incident Cardiovascular Events in a Population-Based Study: The multi-ethnic study of atherosclerosis. Circulation.

[16] Conway J.M., Ingwersen L.A., Moshfegh A.J. (2004). Accuracy of dietary recall using the USDA five-step multiple-pass method in men: An observational validation study. J. Am. Diet. Assoc..

[17] National Academies of Sciences, Engineering, and Medicine (2023). Dietary Reference Intakes for Energy.

[18] Institute of Medicine (2005). Dietary Reference Intakes for Energy, Carbohydrate, Fiber, Fat, Fatty Acids, Cholesterol, Protein, and Amino Acids.

[19] World Health Organization Recommendation for Sugar and Trans Fatty Acids Intake. https://www.who.int/.

[20] DeFronzo R.A., Tobin J.D., Andres R. (1979). Glucose clamp technique: A method for quantifying insulin secretion and resistance. Am. J. Physiol. Endocrinol. Metab..

[21] Friedewald W.T., Levy R.I., Fredrickson D.S. (1972). Estimation of the Concentration of Low-Density Lipoprotein Cholesterol in Plasma, Without Use of the Preparative Ultracentrifuge. Clin. Chem..

[22] Vermeulen A., Verdonck L., Kaufman J.M. (1999). A Critical Evaluation of Simple Methods for the Estimation of Free Testosterone in Serum. J. Clin. Endocrinol. Metab..

[23] Bergman R.N., Finegood D.T., Ader M. (1985). Assessment of Insulin Sensitivity in Vivo*. Endocr. Rev..

[24] American Diabetes Association Diagnosing Diabetes and Learning about Prediabetes. http://www.diabetes.org/diabetes-basics/diagnosis/.

[25] Németh B., Ajtay Z., Hejjel L., Ferenci T., Ábrám Z., Murányi E., Kiss I. (2017). The issue of plasma asymmetric dimethylarginine reference range—A systematic review and meta-analysis. PLoS ONE.

[26] Valenti L., Pelusi S., Bianco C., Ceriotti F., Berzuini A., Prat L.I., Trotti R., Malvestiti F., D’ambrosio R., Lampertico P. (2021). Definition of Healthy Ranges for Alanine Aminotransferase Levels: A 2021 Update. Hepatol. Commun..

[27] Botros M., Sikaris K.A. (2013). The De Ritis Ratio: The Test of Time. Clin. Biochem. Rev..

[28] World Health Organization (2021). Guideline for the Pharmacological Treatment of Hypertension in Adults.

[29] Braunstein G.D., Reitz R.E., Buch A., Schnell D., Caulfield M.P. (2011). Testosterone Reference Ranges in Normally Cycling Healthy Premenopausal Women. J. Sex. Med..

[30] World Health Organization Obesity: Preventing and Managing the global Epidemic. Report of a WHO Consultation. https://apps.who.int/iris/handle/10665/42330.

[31] Paradisi G., Steinberg H.O., Hempfling A., Cronin J., Hook G., Shepard M.K., Baron A.D. (2001). Polycystic ovary syndrome is associated with endothelial dysfunction. Circulation.

[32] Ceriotti F., Henny J., Queraltó J., Ziyu S., Özarda Y., Chen B., Boyd J.C., Panteghini M. (2010). Common reference intervals for aspartate aminotransferase (AST), alanine aminotransferase (ALT) and γ-glutamyl transferase (GGT) in serum: Results from an IFCC multicenter study. Clin. Chem. Lab. Med..

[33] Hoeger K.M., Dokras A., Piltonen T. (2021). Update on PCOS: Consequences, Challenges, and Guiding Treatment. J. Clin. Endocrinol. Metab..

[34] Ramírez-Silva I., Villalpando S., Moreno-Saracho J.E., Bernal-Medina D. (2011). Fatty acids intake in the Mexican population. Results of the National Nutrition Survey 2006. Nutr. Metab..

[35] Vafeiadou K., Weech M., Sharma V., Yaqoob P., Todd S., Williams C.M., Jackson K.G., Lovegrove J.A. (2011). A review of the evidence for the effects of total dietary fat, saturated, monounsaturated and *n*-6 polyunsaturated fatty acids on vascular function, endothelial progenitor cells and microparticles. Br. J. Nutr..

[36] Yang J., Fernández-Galilea M., Martínez-Fernández L., González-Muniesa P., Pérez-Chávez A., Martínez J.A., Moreno-Aliaga M.J. (2019). Oxidative Stress and Non-Alcoholic Fatty Liver Disease: Effects of Omega-3 Fatty Acid Supplementation. Nutrients.

[37] López-Alarcón M.G., Vital-Reyes V.S., Hernández-Hernández F.I., Maldonado-Hernández J. (2020). The role of LCPUFA-ω3 on the obesity-associated hyperandrogenemia of pubertal girls: Secondary analysis of a randomized clinical trial. J. Pediatr. Endocrinol. Metab..

