# Supplementary material for: Obesity and Hyperandrogenemia in Polycystic Ovary Syndrome: Clinical Implications
Source: J Pers Med. 2023 Aug 28;13(9):1319. doi: 10.3390/jpm13091319 (PMC10533085; doi:10.3390/jpm13091319)
Supplement: Supplementary file 1 [file jpm-13-01319-s001.zip › jpm-2516266-supplementary.pdf]

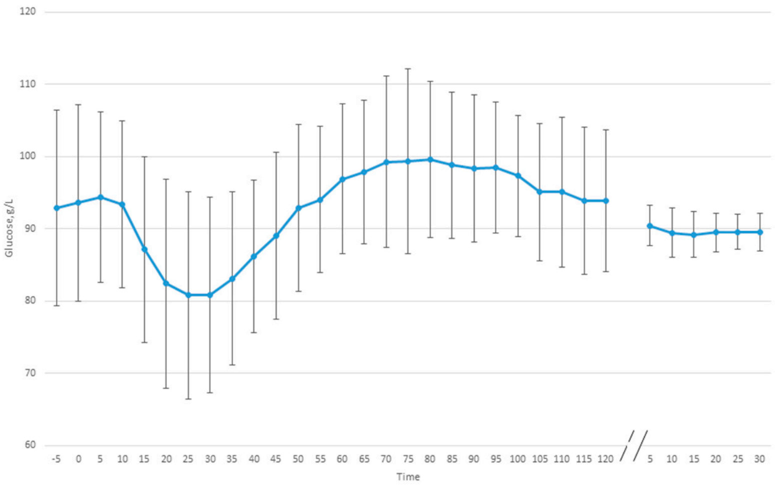

Figure S1. Glucose concentration during clamp procedure. The steady-state was reached at the last 30 min

Table S1 Dietary energy and macronutrient intake

	Absolute values				Recommended*
	Mean ± SE	Median	Q1	Q3	
Energy, kcal/kg	2163 ± 80	1982	1517	2724	2015
Protein, g/d	103 ± 6	87	67	118	46
Carbohydrate, g/d	255 ± 11	234	169	310	130
Fiber, g/d	23 ± 2	17	12	26	25
Sugar, g/d	88 ± 5	76	49	118	25
Total lipids, g/d	71 ± 4	65	39	89	20 - 35
Saturated	22 ± 1	18	12	27	<10
Monounsaturated	20 ± 1	17	9	27	5 – 20
Polyunsaturated	23 ± 9	11	5	20	6 - 11
Trans fatty acids	0.5 ± 0.1	0.2	0.01	0.7	<1
Cholesterol	295 ± 18	224	149	413	0
Omega-3	0.9 ± 0.1	0.6	0.4	1.1	0.6 – 1.2
Omega-6	9 ± 0.6	7	4	12	5 - 10

*References 17-19.

Recommendation of total lipids, saturated, monounsaturated, polyunsaturated fat, and trans fatty acids intake are percentage of daily energy intake. Recommendations of omega-3 and omega-6 fatty acids are expressed as g/d

Table S2. Univariate correlations among studied variables in a sample of 140 women with or without PCOS*

	BMI	FT	M	ADMA	ALT	Non-HDL	CIMT	ALT/AST	FMD
BMI	--								
FT	0.279[a]	--							
M	-0.567[a]	-0.296[a]	--						
ADMA	0.273[a]	-0.030	-0.300[a]	--					
ALT	0.278[a]	0.331[a]	-0.386[a]	0.245[b]	--				
Non-HDL	0.199[c]	0.188[c]	-0.289[a]	0.200[c]	0.320[a]	--			
CIMT	0.163[d]	-0.015	-0.250[b]	0.262[b]	0.047	0.107	--		
ALT/AST	-0.417[a]	-0.317[a]	0.407[a]	-0.238[b]	-0.541[a]	-0.288[a]	-0.044	--	
FMD%	-0.097	0.056	0.200[c]	-0.152[d]	-0.130	-0.059	-0.007	0.107	--
Energy, kcal/d	-0.112	-0.054	0.113	0.050	-0.197[b]	-0.101	-0.051	0.131	0.039
Protein, g/d	-0.171[c]	-0.048	0.107	-0.128	-0.176[c]	-0.096	-0.054	-0.132	-0.064
CHO, g/d	-0.045	-0.042	0.010	0.093	-0.109	0.010	-0.000	0.021	0.036
Fiber, g/d	-0.100	-0.087	0.106	-0.041	-0.88	-0.024	-0.024	0.051	-0.071
Sugar, g/d	-0.079	-0.048	0.000	0.001	-0.075	0.019	-0.115	0.006	-0.069
Total lipids, g/d	-0.078	-0.050	0.152[d]	0.053	-0.70	-0.143[d]	-0.005	0.067	0.007
SAT, g/d	-0.089	-0.045	0.126	0.008	-0.037	0.048	0.004	0.065	0.039
MUS, g/d	-0.104	-0.096	0.195[c]	-0.045	-0.084	-0.007	-0.042	0.122	0.028

PUS, g/d	-0.112	-0.068	0.099	0.044	-0.032	0.091	0.092	0.029	-0.105
Trans fatty acids, g/d	0.036	0.111	0.006	-0.141	0.040	0.101	-0.021	-0.062	0.105
ω-3, g/d	-0.280[a]	-0.090	0.353[a]	-0.104	-0.128	-0.053	-0.103	0.200[c]	-0.033
ω-6, g/d	-0.232[a]	-0.147[d]	0.268[b]	-0.061	-0.156[d]	-0.084	-0.096	0.148[d]	0.040

*Pearson correlation analysis. [a]*p* <0.001, [b]*p* <0.01, [c]*p* <0.05, [d]*p* <0.10

PCOS, polycystic ovary syndrome; BMI, body mass index; FT, free testosterone; M, clam-derived glucose disposal; ADMA asymmetric dimethyl arginine; ALT, alanine aminotransferase; AST, aspartate aminotransferase; HDL, high density lipoproteins; CIMT, carotid intima media thickness; FMD, flow-mediated dilation; CHO, carbohydrates; SAT, saturated fat; MUS, monounsaturated; PUS, polyunsaturated.

Table S3 Clinical and biochemical characteristics of women with or without polycystic ovary syndrome (PCOS).

	PCOS, n = 99	Non-PCOS, n = 41		*P**
	Media ± SEM		Difference ($CI_{95\%}$)	
Age, years	29.2 ± 0.55	29.2 ± 0.84	-0.01 (-2.01, 1.99)	0.993
SBP, mmHg	110 ± 1.1	106 ± 1.8	3.88 (-0.39, 8.15)	0.074
DBP, mmHg	73 ± 0.98	71 ± 1.3	2.6 (-0.61, 5.86)	0.111
BMI, kg/m^2	31.8 ± 0.59	27.2 ± 1.0	4.57 (2.3, 6.9)	<0.001
Body fat, %	42.4 ± 0.66	38.1 ± 1.3	4.27 (1.35, 7.2)	<0.001
Glucose, mg/dL	88 ± 1.1	84.8 ± 1.4	3.17 (-0.27, 6.61)	0.071
Insulin, µU/mL	16.4 ± 1.3	7.5 ± 0.88	8.9 (5.8, 12.0)	<0.001
TC, mg/dL	167 ± 3.4	156 ± 5.1	11.1 (-1.1, 23)	0.074
TG, mg/dL	153 ± 8.8	124 ± 11	29 (1.0, 56)	0.043
VLDL, mg/dL	30.5 ± 1.8	24.3 ± 2.1	6.16 (0.7, 11.7)	0.028
HDL, mg/dL	38 ± 0.94	42.4 ± 1.6	-4.5 (-8.16, -0.84)	0.017
LDL, mg/dL	99 ± 3.0	89 ± 4.2	10.25 0.08, 20.4)	0.048
Non-HDL, mg/dL	130 ± 3.5	113 ± 5	16.7 (4.52, 28.9)	0.008
SHBG, mmol/mL	28.2 ± 1.6	42.2 ± 3.4	-14.1 (-21.6, -6.6)	<0.001

M, mg/kg/min	4.82 ± 0.27	7.8 ± 0.56	-2.95 (-4.2, -1.7)	<0.001
ALT, U/L	40.5 ± 3.3	24.8 ±2.3	15.64 (7.8, 23.5)	<0.001
AST, U/L	37.3 ± 2.1	29.8 ± 1.5	7.48 (2.45, 12.5)	<0.001
GGT, U/L	30.1 ± 2.1	22.5 ± 3.1	7.56 (0.11, 15.0)	0.047
AST/ALT	1.06 ± 0.04	1.35 ± 0.07	-0.28 (-0.43, -0.13)	<0.001
TT, ng/dL	38.1 ± 2.4	26.8 ± 2.6	11.2 (4.2, 18.3)	0.002
FT, pg/mL	8.2 ± 0.63	4.6 ± 0.60	3.2 (1.6, 4.9)	<0.001
ADMA, µmol/L	1.13 ± 0.04	1.01 ± 0.04	0.11 (0.02, 0.21)	0.024
CIMT, mm	0.61 ± 0.02	0.56 ± 0.03	0.05 (-0.02, 0.12)	0.068
FMD, %	21.0 ± 1.6	23.1 ± 3.2	-1.92 (-9.11, 5.16)	0.311

*Student t-test.

SBP, systolic blood pressure; DBP, diastolic blood pressure; BMI, body mass index; TC, total cholesterol; TG, triglycerides; VLDL, very low density lipoprotein, HDL, high density lipoprotein; LDL, low density lipoprotein; SHBG, steroid hormone binding globulin; ALT, alanine aminotransferase; AST, aspartate aminotransferase; GGT, gamma glutamyl transferase; TT, total testosterone; FT, free testosterone; ADMA asymmetric dimethyl arginine; CIMT, carotid intima media thickness; FMD, flow mediated vasodilation.
